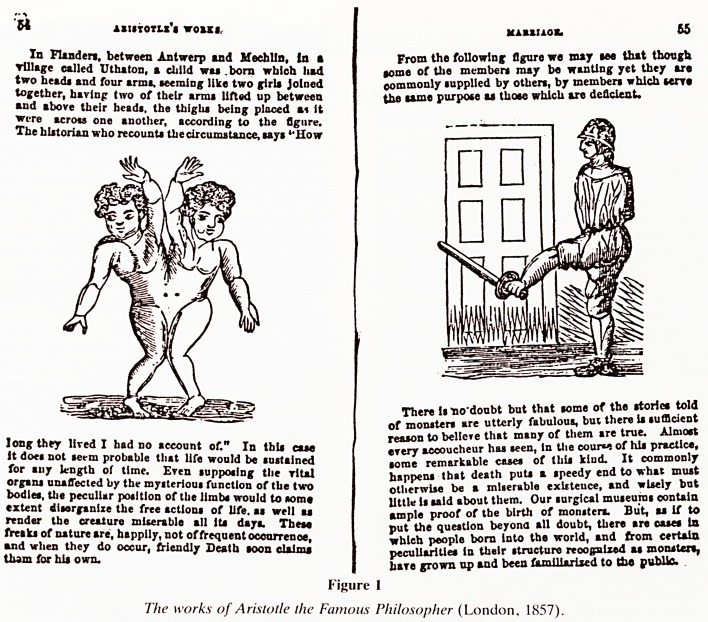# The Medicine of the People

**Published:** 1990-06

**Authors:** Allan Chapman

**Affiliations:** Centre for Medieval and Renaissance Studies, Oxford


### The Medicine of the People; the survival of classical medical ideas into modern popular usage

**Dr Allan Chapman**
Centre for Medieval and Renaissance Studies, Oxford

No human activity with a lineage spanning many centuries can avoid the accumulation of apparent archaisms. Medicine, like language, is rich in them, and though they may be redundant from the standpoint of contemporary scientific practice, they nonetheless tell us much about origins and developments. When one thinks of the language in which we describe medical states, we find an avenue which not only illuminates a great deal about the history of illness, but which provides some clues whereby we can understand the way in which many patients still envisage it today.

We still encounter people who lack "vital spirits", possess "warm hearts" and "cool heads", and whose blood "runs cold" in old age. Victorian patent medicine manufacturers and modern naturopathic healers alike place great stress on the liver and the quantity of blood which it "throws". Anger is concomitant with an excess of "spleen", while bile and phlegm are attributed with their own pathological states. A Somerset quack was prosecuted in 1875 for treating a consumptive's "dry liver" with a miscellany of herbs intended to generate new blood (1), while a lady of my own acquaintance was told in 1987 by a fashionable naturopath that her cough was "rooted" in her liver and its bad blood! The treatment prescribed to the Victorian consumptive and the modern housewife were astonishingly similar; herbs which attracted the "warmth" of the sun, to be taken at astrologically determined times. Ancient explanations die hard, especially when re-dressed in the garments of contemporary prosperity.

For many years, as a medical historian, I have collected descriptions and explanations of disease encountered in modern popular usage. These include both the spoken and written word, along with the publicity language of modern and Victorian fringe medicine. Though the modern person has a vague familiarity with what contemporary medicine attempts to do, it is often seen in the context of wonder drugs and daring surgical procedures. On the explanatory level, however, many ordinary and not very well educated people lapse back into a world of semi-vital forces, with "predatory" diseases caused by stinks or bad air, that would not have been unfamiliar to Hippocrates or Aristotle.

I first came to be fascinated by these "archaisms" when I heard them articulated by my own grandparents and other surviving Victorians in my family in Lancashire. Then, I began to ask them what they thought certain diseases were, and widened my inquiries first to their friends of the same generation, and on to a substantial cross section of old people. Upon request, medical practitioners, mainly in the Manchester and Liverpool areas, began to supply me with examples of how patients in the consulting room described their own infirmities, from which it was possible to re-construct a remarkable medical sub-culture. It is in no way my argument, of course, that this sub-culture possesses any clearly thought out or conscious rationale, though it does contain—in its stress on good and bad, hot and cold, blood, phlegm and spleen—fragments of a system of medicine which long ante-dates anything taught in a modern medical school.

These are the elements of the "system". Health exists when all things within the body are in a state of balance. Disease is caused by an imbalance of vital forces, usually of blood. Phlegm, blood, bile and spleen produce hot, cold, moist and dry symptoms by their excess or deficit. Too much "hot" blood, for instance, causes feverishness, while the thin, cold blood of the elderly occasions feebleness owing to its lack of nourishment. Pneumonia and bronchitis are caused by "cold" phlegm. Life is associated with radiant heat, death with cold coagulation. Disease can be envisaged as a malign, predatory agency waiting to pounce on its victim and upset his balance. People "go" mad, "have the cancer", and "get bad hearts". Cold diseases are cured by warm remedies, and *vice versa*.

Life, in short, resembles a game of chance and avoidance, in which the malign forces of disease must be outwitted by the cunning foresight of patient and doctor.

It is, indeed, the rationale of classical medicine, as encapsulated in the Hippocratic *Corpus*, the physiology of Aristotle and the writings of Galen (2). It is rooted in the humoral tradition which formed the basis of Christian (and Muslim) academic medicine from antiquity to the seventeenth century, where balance, blood, phlegm and bile provided the explanations for health and disease; where symptoms of illness were treated by "contrary" therapies, and the right foods were seen as essential to generating the right humours.

This concentration of health and welfare formed the basic curriculum of the medieval medical schools from the thirteenth century, amplified by the Latin commentaries such as the *Dietry* and *Regimen* of Salerno (3). But the tradition was essentially an academic one, and I became fascinated in trying to trace a route whereby it entered popular, vernacular culture, and came to survive there virtually intact, into the twentieth century.

I believe it was in Tudor England that the tradition first came to be popularised in a series of works purporting to teach "poor men's physick". The *Dyetry* (1542) and *Breviary of Helthe* (1547) of Dr Andrew Boorde overtly claimed to bring physic within the reach of the poor. William Bullein, Thomas Brassbridge and a variety of medical graduates produced vernacular treatises on general and specific illnesses over the next forty years, backed by medical botanists like John Gerard, whose great *Herball* (1597 and 1633) considerably amplified the tradition (4). Why this sudden appearance of academic medicine in vernacular editions took place is not clear, but one suspects that growing literacy, the Reformation suppression of many charitable institutions and the cheaper cost of printing all contributed, alongside a broader humanitarian concern (5).

But the Tudor poor were not generally book purchasers. It is my suspicion, however, that these vernacular medical digests received further popularisation in the hands of the Almanack compilers whose tuppenny and threepenny astrological creations invariably carried medical sections in their thirty-odd pages of tiny print. Some Almanack writers even claimed formal medical qualifications, such as John Securis of Salisbury, who in his 1579 *Almanack* styled himself "Master of Art and Physike" (6).

Almanacks, in many ways, constituted the bedrock of the printed word in the sixteenth century, and we have plenty of evidence of their popularity amongst the poor. They were read aloud around the winter fireside, while a literate artisan, like Quince the Joiner in Shakespeare's *Midsummer Night's Dream*, had one in his pocket when the Mechanicals were looking for a moonlit night on which to rehearse their play (7). By the mid-seventeenth century, there were many avenues through which the broader precepts and many of the details of classical academic medicine had become available to simple English readers, culminating, perhaps, in Nicholas Culpeper's *English Physitian* (1652), which claimed to lay bare the secrets of the Latin *Pharmacopoeia* to the common man (8).

Ironically, though, while this process was giving the English reader access to the already 2,000 year old classical medical tradition, the most advanced medical thinkers of the seventeenth century were turning to pastures new in their attempts to understand disease. Impressed by the triumphs of the "New Science" in astronomy, mechanics and physics, the Mechanical Philosophy of experimentation was seen as possessing a relevance to medicine.

The Paduan anatomists, after Vesalius, were increasingly looking to systematic dissection as a guide to bodily function. Sanctorius recognised the importance of weight change and measured the pulse rate to try to establish their relation to health, while Descartes, Glisson and others began to study muscular action in mechanical terms. In the wake of the new movement came William Harvey's announcement in 1628 of the blood's *circulation* throughout the body. Harvey's followers over the next forty years demonstrated his discovery with the microscope, and undermined the classical view which claimed that blood was a "humour" generated in the liver from food, heated in the heart, and sent effervescing into the *veins* on a one-way journey, to be turned into flesh, skin and body heat (9).

Gradually, physicians came to see the heart as a pump, rather than a crucible, while Harvey's disciples such as Mayow, Willis and Bartholin had come close to explaining the relationship between the blood, lungs and lymph system by 1680. By this time, indeed, the body was coming to be envisaged in academic medical circles as "physico-mechanical", in which respiration was seen as a chemical process, mental activity as a "hydraulic" one, as fluids moved between the chambers of the brain, and the action of the muscles and skeleton as analagous to the parts of a watch (10). Though not a physician, this new sense of the "man machine" was most aptly captured by Thomas Hobbes in the Introduction to *Leviathan* (1651), by saying;

> "For what is the *Heart*, but a *Spring*; and the *Nerves* but so many *Strings*; and the *Joynts*, but so many *Wheeles*,
> giving motion to the whole Body, such as was intended by the Artificer".

Hobbes, who was a friend of William Harvey, was undoubtedly an advanced thinker to conceive of man as an "*Automata*" at this time, but he indicated a radically different stance from that of classical medicine. In practical, therapeutic terms, however, the New Science had little to offer, and the most "philosophical" of physicians could do precious little for their patients. What was going from academic medicine, though, was the classical view of the body as a collection of imponderable vital forces.

Yet there is no evidence that any of these developments had any impact on popular medical understanding, where disease still continued to be seen in the composite terms of religious retribution, magical affliction *and* simplified classical physiology. This gulf between how the academic physician and the common man viewed disease was made explicit, indeed, by John Wesley in 1747. In his *Primitive Physick* of that year, Wesley castigated scientific medicine for its pursuit of the speculative at the expense of the relief of the poor. According to Wesley, medicine was intended as a gift from God to mitigate disease caused by original sin;

> "But in the process of time, men of philosophical turn were not satisfied with this ... They examined the human body in all its parts; the nature of the flesh, veins, arteries, nerves ... brain, heart, lungs ... to set physic upon hypothesis. As theories increased, simple medicines were more and more disregarded and disused till in a course of years the greater part of them were forgotten, at least in the politer nations" (11).

By 1747, therefore, one can discern a clear parting of the ways, between an already well established tradition of scientific, experimental medicine, and the medicine of the people. *Primitive Physick* was a book of simple "cures" and homely remedies, and while it was not systematically classical, nonetheless shares with Boorde and the Almanack writers the desire to break down professional exclusiveness, and give relief to the poor in a way which they could understand. Considering the enormous impact of Wesley as a religious leader, and the deep inroads which Methodism made into the ranks of the poor, one can understand how *Primitive Physick* contributed in confirming certain popular attitudes towards illness.

Another work which was to have a massive impact in this respect, especially among women, was the pseudo *Aristotle's Works* (12). Anonymously compiled from the *De Generatione Animalium*, Book II, the *Historia Animalium* (13), and other physiological writings of Aristotle, probably in the seventeenth century, the *Works* enjoyed a great vogue in Victorian and Edwardian England, as a manual on sex, pregnancy and children. It was coyly referred to as a "Housewife's book", and purported to answer a wide range of questions upon an unmentionable subject.

Babies were conceived when the male "seed" thickened in the menstrual blood, (like the curdling of rennet to form cheese) (14). Boys were conceived in the right hand "horn" of the womb and girls in the left. A pregnant woman frightened by an animal could bear a child which resembled the animal, as in the case of the hideously deformed Joseph Merrick—the "Elephant man"—whose mother had been frightened by a circus elephant while pregnant (15). The *British Library Catalogue* records twenty-two British editions of the *Works* between 1777 and 1905 (16). Gracie Fields, listing the items necessary for a hopeful spinster's trousseau in her song "My little bottom drawer" in 1934, mentions the *Works* (17), while an Oxford colleague still recalls copies on sale in a Bristol newsagent's shop in the 1940s. He mentioned that he, and his fellow schoolboys, used to sneak in when the proprietor was occupied and pore over the lurid contents of "the philosopher" until they were detected and chased out.

While it is true that the precise physiology of mammalian reproduction remained something of a mystery until the late nineteenth century, *Aristotle's Works* were still hopelessly out of date by 1900, and would have passed on and confirmed many classical ideas to the working classes, to enrich their stock of "old wives' tales". I still remember, as a small child in the mid 1950s, hearing my grandmother's friends referring to "Harry Stotle" and grinning about his "work". Only later, did these remembered phrases from my childhood memory take on any meaning to me, and when as a student, I recognised them as living fragments of antiquity. In later years, when ladies of that generation were in the eighties and nineties, and I was researching the subject, I found that the mention of Aristotle would produce an embarrassed silence—at least before a male questioner.

Another avenue through which this tradition drew its strength and confirmation was Victorian and Edwardian quackery. In the fiercely competitive world of quack nostrums, it was essential that a successful preparation should fulfil certain criteria to attract purchasers. Cough medicines had to be thick, warm and viscous if they were going to "cut

54 ARISTOTLE'S WORKS.

In Flanders, between Antwerp and Mechlin, in a village called Uthaton, a child was born which had two heads and four arms, seeming like two girls joined together, having two of their arms lifted up between and above their heads, the thighs being placed as it were across one another, according to the figure. The historian who recounts the circumstance, says "How long they lived I had no account of." In this case it does not seem probable that life would be sustained for any length of time. Even supposing the vital organs unaffected by the mysterious function of the two bodies, the peculiar position of the limbs would to some extent disorganize the free actions of life, as well as render the creature miserable all its days. These freaks of nature are, happily, not of frequent occurrence, and when they do occur, friendly Death soon claims them for his own.

MARRIAGE. 55

From the following figure we may see that though some of the members may be wanting yet they are commonly supplied by others, by members which serve the same purpose as those which are deficient.

There is no doubt but that some of the stories told of monsters are utterly fabulous, but there is sufficient reason to believe that many of them are true. Almost every accoucheur has seen, in the course of his practice, some remarkable cases of this kind. It commonly happens that death puts a speedy end to what must otherwise be a miserable existence, and wisely but little is said about them. Our surgical museums contain ample proof of the birth of monsters. But, as if to put the question beyond all doubt, there are cases in which people born into the world, and from certain peculiarities in their structure recognized as monsters, have grown up and been familiarized to the public.

**Figure 1**

*The works of Aristotle the Famous Philosopher* (London, 1857).

the phlegm" and "warm the lungs", thereby driving out cold. Purges—so essential to a constipated population—must "drive out all poisons", while "Little Liver Pills", "Blood Purifiers" and "Bile Beans" catered for people who *knew* which of their "organs" was at fault. Advertisements in the popular newspapers of the period make it clear that neither the scientific revolution nor the massive developments in nineteenth century medicine had much impact on the popular consciousness, which still thought in basically Galenic terms (18).

One reason for this conservatism was the primitive level of general education at the turn of the century, while another stemmed from the financial inaccessibility of the medical profession. A month's supply of a quack nostrum could be bought for the same sum as a consultation with a qualified doctor, not to mention the additional cost of his prescription. Yet another arose from the deeply held folk belief in self-medication and distrust of experts. Though not especially classical in origin, one must never lose sight of the Hippocratic tradition's largely commonsensical approach to disease, and John Wesley's castigation of the exclusiveness of doctors.

But with the passing of Lloyd George's Health Insurance Act in 1911, at least breadwinners could get access to qualified doctors, and within a few years, the rest of their families (19). With the creation of the National Health Service in 1948, one might have expected the death-knell of ancient medicine to have been rung, though such an expectation still fails to reckon with two powerful elements in the popular tradition; the love of self-medication, and belief in the supreme efficacy of simple cures.

Nowhere have these two elements more successfully mushroomed during recent years than in alternative medicine. Popular books, night-school classes and even chainstore herbalists—most noticeably "Culpeper the Herbalist"—resurrect the old teaching. Good health is simple balance; disease is caused by some sort of poison or stress, scientific medicine is too impersonal and "chemical", while a wise person follows "mother nature". Shrewdly marketed, in aroma-laden shops quaintly decorated and drawing heavily upon nostalgia, the medicine of the people has become big business. Advised, no doubt, by good lawyers, no *cures* as such are promised, but both products and promotional literature emphasise the general healthiness which one will feel by following the firm's guide to mother nature. Though mercifully freed from *Aristotle's Works*, I have met people under forty whose knowledge of phlegms, bad blood and vital heat could rival that of my grandmother, plus an additional eclectic knowledge of germs, vitamins and antibiotics.

Yet truly astonishing, is the persistence with which the *rationale* of classical medicine has taken hold of the imagination. Chameleon-like, it has migrated from the ancient world, through the medieval universities, the peasant's cottage, the industrial slum, and now, into the fashionable suburban apartment. It has moulded our language about how we think of the disease process, tells us much about our instinctive reactions to the natural world, and provides us with a filament linking our world with one of the most remarkable achievements of classical civilization.

### NOTES AND REFERENCES

1. Walter Rivington, *The Medical Profession*, (Dublin, 1879) 93.
2. The principal texts are reproduced in the *Hippocratic Writings*, transl., J. Chadwick & W. N. Mann, Penguin Classics (Harmondsworth, 1983). Aristotle, G ⅞ nimalium: Historia Animalium: & De Generatione Animalium, in *The Works of Aristotle*, Vols. IV & V (Oxford, 1912 & 1910). Galen, *On the Natural Faculties*, transl., A. J. Brock, Encyclopaedia Britannica Inc., Harvard Univ. Press (1952).
3. No modern edition, see Thomas Paynel's translation, *Regimen Sanitas Salerni*, (London, 1541). Later English translations available i.e. (London, 1617). Stanley Rubin, *Medieval English Medicine*, (Newton Abbott, 1974).

Lecture delivered to Bristol Medico-Chirurgical Society on Jan. 10th 1990.

4. See, Paul Slack, "Mirrors of health and treasures for poor men", in Charles Webster, Ed., *Health, Medicine and Mortality in the Sixteenth Century*, (Cambridge, 1979) 237–273.
5. Eminent contemporaries such as Sir Thomas More and modern historical researchers agree that literacy, in some form, touched about 45% to 50% of Tudor people; Antonia MacLean, *Humanism and the Rise of Science*, (London, 1972) 82.
6. Allan Chapman, "Astrological Medicine", in Webster, op. cit., 275–300. John Securis's *Almanack* for 1579 is in the Bodleian Library, Ashmole 62. The Bodleian contains one of the finest collections of 16th–17th century Almanacks extant.
7. *Midsummer Night's Dream*, Act III, Scene 2.
8. Culpeper retained great popularity. I possess a copy printed in Manchester in 1826, while modern reprints are often on sale in 'natural health' shops. See also, Agnes Arber, *Herbals* (1912).
9. Charles Singer & E. A. Underwood, *A Short History of Medicine* (Oxford, 1962) 59–66.
10. Allan Chapman, "Mind, Body and Brain; Medicine and the Mechanical Philosophy in seventeenth century Europe", Hastings Memorial Lecture, Dept. of Psychiatry, University of Minnesota, 1982. Paper circulated by Hastings Memorial Committee.
11. John Wesley, *Primitive Physick, or a short and easy way of curing most diseases*, (London, 1747), cited from 5th Edition, (Bristol, 1755) Paras. 8–9 in "Preface".
12. *The Works of Aristotle the Famous Philosopher*; numerous editions were printed, often undated on cheap paper. I have a well-produced London edition of 1857. The Radcliffe Science Library, Oxford possesses two undated copies of *c*.1905.
13. Janet Blackman, "Popular theories of generation; the evolution of *Aristotle's Works . . .* ", in *Health care and popular medicine in nineteenth century England*, Ed., J. Woodward and D. Richards (London, 1977) 56–88.
14. *Aristotle's Works* usually broke down into four sections. Most of the material on reproduction came from the sections "Aristotle's Masterpiece" and "Problems".
15. Michael Howell and Peter Ford, *The True History of the Elephant Man*, (London, 1983) 30, 223. Joseph Merrick (the "Elephant Man") always insisted that his deformities arose after his pregnant mother was frightened by a circus elephant, repeating the story to Sir Frederick Treves and others. The association of deformity with an object of fear possessed a deeply classical connotation, and was discussed in the "Marriage" section of *Aristotle's Works*, where women were advised against shocks in pregnancy and the "monsters" which they could bring forth after receiving them.
16. Of these 22 editions, only 12 carried an exact publication date, the rest being dated by internal evidence, such as typeface, testimonial letters *et cetera*. Considering the 'under the counter' character of the *Works*, it is likely that many other unrecorded editions were printed.
17. "In my little bottom drawer", written by Will E. Haines and Jimmy Harper, was sung in the film *Sing as we go* (1934). Because of the song's continuing popularity after *Aristotle's Works* had declined in familiarity to later audiences, it was changed to "Priestley's Works" (J.B.?). The authentic words are printed in the text of *Our Gracie* (1978) E.M.I. Music Publishing Co.
18. The B.M.A. publication *Secret Remedies, what they cost and what they contain*, (London, 1909) was intended to expose many quack "cures". It also printed the claims of the preparations, as they appeared on the bottle labels, which often indicate their supposed action upon the blood and liver.
19. John Cule, *A Doctor for the People*, (London, 1980) Chapter 14.

## Figures and Tables

**Figure 1 f1:**